
JOURNAL INFORMATION
==============================
NLM Title Abbreviation: Biospektrum (Heidelb)
Journal ID: BIOspektrum (Heidelb.)
NLM Journal ID: 9615685

Biospektrum

ISSN: 0947-0867
EISSN: 1868-6249

ARTICLE INFORMATION
==============================
PMCID: PMC7318729
PMID: 32834541
DOI: 10.1007/s12268-020-1396-0
Article ID: 1396
Article version: 1
Subjects: Karriere, Köpfe & Konzepte

Gemeinschaftlich in Krisenzeiten: NMR-Strukturbiologie gegen COVID-19

Schlundt A.
Wirtz M. A.
Knezic B.
Hengesbach M.
Fürtig B.
Weigand J. E.
Wöhnert J.
Ferner J.
Saxena K.
Wacker A.
Richter C.
Sreeramulu S.
Wirmer-Bartoschek J.
Schwalbe Harald schwalbe@nmr.uni-frankfurt.de http://schwalbe.org.chemie.uni-frankfurt.de

Im Namen von Mehr als 50 Wissenschaftlerinnen und Wissenschaftlern in Frankfurt und Darmstadt
Institut für Organische Chemie und Chemische Biologie, Zentrum für Biomolekulare Magnetische Resonanz (BMRZ), Universität Frankfurt a. M., Max-von-Laue-Straße 7, D-60438 Frankfurt a. M., Deutschland
Electronic publication date: 2020 Jun 26
Print publication date: 2020
Volume: 26
Issue: 4
First page: 440
Last page: 441
Copyright: © Die Autoren 2020
License: Open Access: Dieser Artikel wird unter der Creative Commons Namensnennung 4.0 International Lizenz veröffentlicht, welche die Nutzung, Vervielfältigung, Bearbeitung, Verbreitung und Wiedergabe in jeglichem Medium und Format erlaubt, sofern Sie den/die ursprünglichen Autor(en) und die Quelle ordnungsgemäß nennen, einen Link zur Creative Commons Lizenz beifügen und angeben, ob Änderungen vorgenommen wurden. Die in diesem Artikel enthaltenen Bilder und sonstiges Drittmaterial unterliegen ebenfalls der genannten Creative Commons Lizenz, sofern sich aus der Abbildungslegende nichts anderes ergibt. Sofern das betreffende Material nicht unter der genannten Creative Commons Lizenz steht und die betreffende Handlung nicht nach gesetzlichen Vorschriften erlaubt ist, ist für die oben aufgeführten Weiterverwendungen des Materials die Einwilligung des jeweiligen Rechteinhabers einzuholen. Weitere Details zur Lizenz entnehmen Sie bitte der Lizenzinformation auf http://creativecommons.org/licenses/by/4.0/deed.de.
License URL: https://creativecommons.org/licenses/by/4.0/

issue-copyright-statement: © Springer-Verlag GmbH Deutschland, ein Teil von Springer Nature 2020

==============================

COVID19-NMR: A. SCHLUNDT, M. A. WIRTZ, B. KNEZIC, M. HENGESBACH, B. FÜRTIG, J. E. WEIGAND, J. WÖHNERT, J. FERNER, K. SAXENA, A. WACKER, C. RICHTER, S. SREERAMULU, J. WIRMER-BARTOSCHEK, H. SCHWALBE
IM NAMEN VON MEHR ALS 50 WISSENSCHAFTLERINNEN UND WISSENSCHAFTLERN IN FRANKFURT UND DARMSTADT

■ Seit Ende 2019 hält uns SARS-CoV-2 (*severe acute respiratory syndrome-related coronavirus 2*) in Atem. Seit Anfang dieses Jahres gibt es die ersten Sequenzinformationen über das Genom des Virus, das als Auslöser der Lungenerkrankung COVID-19 gilt (*coronavirus disease 2019*). Aus diesen Daten geht hervor, dass das Betacoronavirus SARS-CoV-2 mit seinen fast 30.000 Einzelstrang-RNA-Nukleotiden für ungefähr 30 Proteine codiert. Es besitzt sehr hohe Sequenzhomologie mit dem bereits 2002 in Asien aufgetauchten SARS-CoV der ersten Generation. Sowohl die Proteine als auch die RNA des Virus stellen über ihre 3D-Struktur molekulare Ziele für eine pharmakologische Intervention dar.

### Das Konsortium COVID19-NMR

Mitglieder des Sonderforschungsbereichs 902 „Molekulare Prinzipien der RNA-basierten Regulation“ haben sich daher entschieden, sämtliche RNAs, die als potenziell regulatorische Einheiten diskutiert werden, sowie alle codierten Proteine in dem Projekt „COVID19-NMR“ mittels kernmagnetische Resonanzspektroskopie (*nuclear magnetic resonance* (*NMR*) *spectroscopy*) zu untersuchen. Ziel ist es, systematisch die NMR-Signale der Proteine und RNAs zuzuordnen und relevante Strukturen zu bestimmen. Diese Daten liefern die Grundlage, um Liganden niederen Molekulargewichts auf ihre Bindung an virale Zielproteine und Ziel-RNAs zu untersuchen. Mittels NMR können Substanzbibliotheken von vielen Hunderten Liganden schnell untersucht werden. Dabei kann die NMR-Spektroskopie sowohl über den Ort, die Affinität und die Kinetik der Ligandenbindung wertvolle Aussagen treffen.

Das COVID19-NMR-Konsortium hat Ende März seine Arbeit aufgenommen. Sechs Gruppen von der Goethe-Universität Frankfurt und der TU Darmstadt haben sich zusammengetan. Von Anfang an galt, so schnell wie möglich so viele Proteine und RNA-Elemente zu untersuchen wie kapazitiv machbar, und die Ergebnisse dieser Untersuchung sofort der wissenschaftlichen Gemeinschaft über das Internet frei zugänglich zu machen (covid19-nmr.de). Aus der Initiative des SFB902 ist mittlerweile ein internationales Konsortium mit mehr als 30 teilnehmenden Laboren aus 11 Ländern entstanden, das schnell erste Erfolge verzeichnete (**Abb. 1**). Der Ansatz ist vor allem in der Breite auf RNA-Ebene einzigartig.

### RNA-Strukturbiologie: die Strategie

Wir haben die regulatorisch aktiven Bereiche in den 5’- und 3’-untranslatierten Regionen (UTRs) sowie einen für ein *frameshifting* verantwortlichen Pseudoknotenbereich in der RNA einzeln kloniert und untersuchen diese Konstrukte sukzessive hinsichtlich ihrer Struktur. Dazu haben wir zunächst 15 verschiedene RNA-Zielmoleküle definiert. Bereits nach einem Monat Arbeit

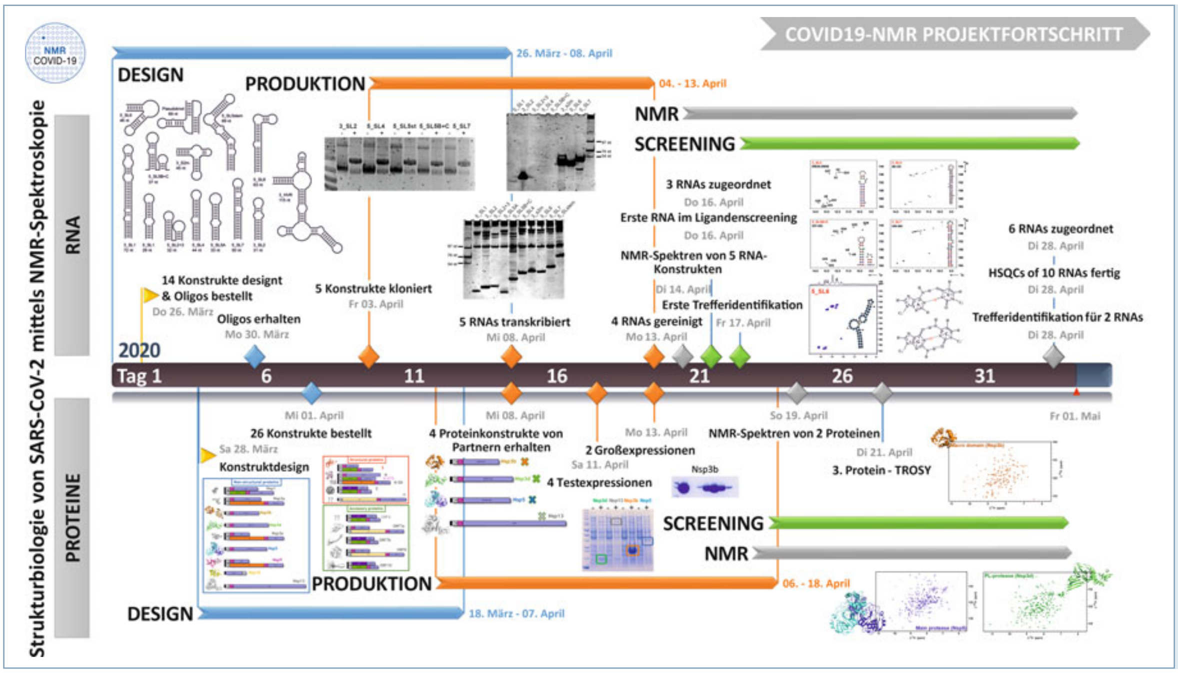

◀ **Abb. 1:** Zeitliche Übersicht über die Fortschritte im COVID19-NMR-Projekt innerhalb des ersten Monats kurz nach Ausbruch der Epidemie in Europa. Von Ende März bis Ende April 2020 gelang unter anderem der komplette Weg vom Konstruktdesign zur Identifizierung von Treffern in einem Screen gegen SARS-CoV-2-RNA-Strukturen. Abbildung: Heidi Zetzsche.

Springer

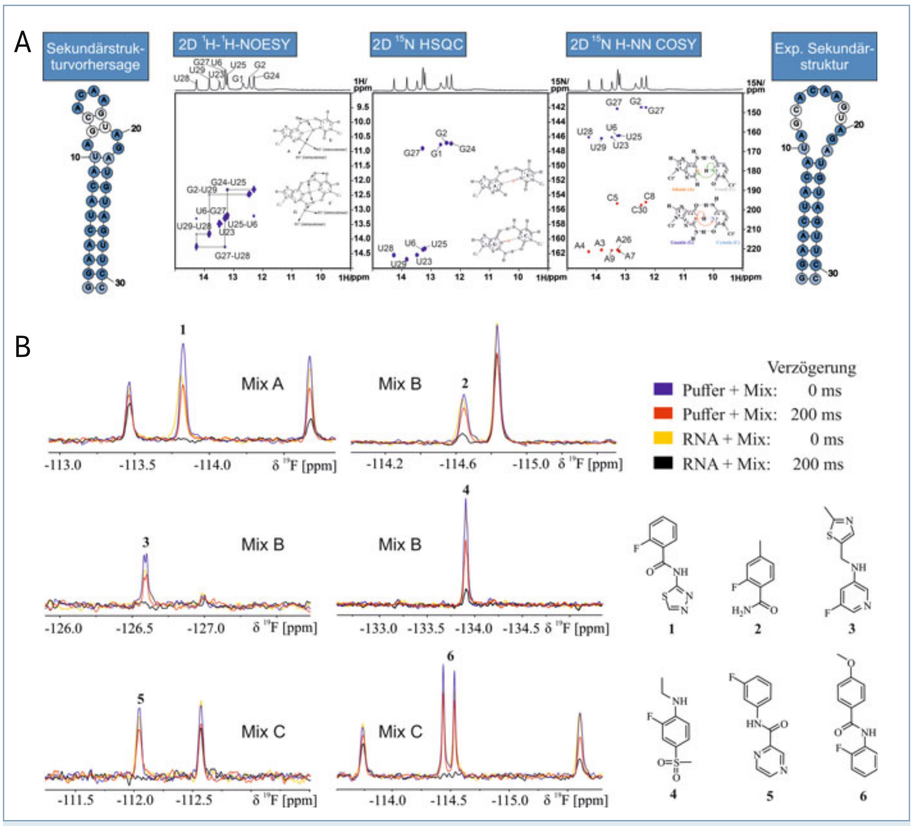

▲ **Abb. 2:** NMR-basiertes Ligandenscreening von RNAs. **A**, NMR an einem Element aus dem 5'-UTR des SARS-CoV-2-RNA-Genoms. Links: Sekundärstrukturvorhersage. Mittels verschiedener 2D-Experimente können die einzelnen Basenpaare detektiert und Strukturelemente eindeutig nachgewiesen werden. Die experimentell ermittelte Sekundärstruktur (rechts) stellt den Ausgangspunkt für das Screening von Substanzen dar. **B**, Links: NMR-Signatur des Screenings von Substanzbibliotheken an eine strukturierte RNA aufgrund der unterschiedlichen Höhe der $^{19}$F-Signale. Rechts: die chemischen Strukturformeln der bindenden Liganden.

haben wir für neun dieser Konstrukte sehr gute Spektren erhalten, die die Faltung der RNA verraten, und die NMR-Signale für acht RNAs bereits zuordnen können. Diese bis hierhin einzelnen RNA-Elemente werden nach und nach in ihrem jeweils größeren Sequenzzusammenhang analysiert, um die oft komplexe Struktur von RNAs korrekt zu erfassen.

Im nächsten Schritt testen wir Ligandenbibliotheken auf Bindung an diese RNA-Elemente. Bisher finden wir eine Anzahl von selektiv einzelne RNAs bindenden Liganden. Die besten dieser Liganden werden dann von Kollegen funktional auf virale Inhibition untersucht. Umgekehrt erhalten wir die besten Hits aus biologischen Screens, um dafür das molekulare Target, sei es RNA oder Protein, zu identifizieren.

## Was kann die NMR-Spektroskopie dazu leisten?

NMR-spektroskopische Untersuchungen von RNAs starten mit der Herstellung von einigen Milligramm einer RNA-Probe mittels T7-Polymerase-katalysierter *in vitro*-Transkription. Wir nutzen dazu Ribonukleosidtriphosphate (rNTPs), die mit nicht-radioaktive, NMR-aktiven Isotopen $^{15}$N- und auch $^{13}$C-markiert sind. Mittels Analyse mehrdimensionaler Spektren, die an auf diese Weise markierten RNAs aufgenommen werden, können die NMR-Signale zugeordnet werden. NMR-Spektroskopie kann dabei jedes einzelne Atom auflösen (**Abb. 2A**).

Diese Zuordnung der NMR-Signale bildet die Basis dafür, verfolgen zu können, an welchem Ort und mit welcher Affinität ein Ligand an eine RNA bindet. Denn die chemische Verschiebung (sozusagen die Position im Spektrum) oder die Intensitäten der Signale verändern sich, wenn der Ligand die Konformation in der Bindungstasche einer RNA, oder aber auch eines Proteins, variiert. Dies wird mittels NMR im Zielmolekül atomar aufgelöst sichtbar, was einen der großen Vorteile der NMR-Methodik darstellt.

In neuerer Zeit zeigt sich, dass auch RNAs durch kleine Moleküle gezielt gebunden und in ihrer Funktion inhibiert werden können. Deshalb untersuchen wir die RNA auf ihre Bindung an potenzielle Wirkstoffe. Typischerweise screenen wir Substanzbibliotheken mit bis zu 768 Liganden. Diese Substanzbibliotheken sind vorab auf chemische Diversität ebenso optimiert worden wie auf die prinzipielle Möglichkeit, sie chemisch einfach zu modifizieren. Die experimentellen Daten eines solchen Screenings zeigt beispielhaft **Abbildung 2B**. Hier wurde in mehreren Mischungen mit jeweils etwa 20 Verbindungen, die ein NMR-aktives $^{19}$F-Atom besitzen, die Bindung an eine strukturierte RNA untersucht. Im Kontext von COVID-19 zeigt sich hier ein enormer Vorteil unserer Methodik: Die deutlich reduzierte spektrale Komplexität von Fluor-NMR-Spektren des Liganden beschleunigt die Auswertung eines Screenings.

Über den systematischen Ansatz von Screens an allen ermittelbaren RNA- und Proteinstrukturen von SARS-CoV-2 erwarten wir einen entscheidenden Beitrag zur langfristigen Therapierbarkeit von COVID-19 sowie von möglichen zukünftigen Epidemien, die durch Coronaviren ausgelöst werden, leisten zu können. ■

**Open Access:** Dieser Artikel wird unter der Creative Commons Namensnennung 4.0 International Lizenz veröffentlicht, welche die Nutzung, Vervielfältigung, Bearbeitung, Verbreitung und Wiedergabe in jeglichem Medium und Format erlaubt, sofern Sie den/die ursprünglichen Autor(en) und die Quelle ordnungsgemäß nennen, einen Link zur Creative Commons Lizenz beifügen und angeben, ob Änderungen vorgenommen wurden. Die in diesem Artikel enthaltenen Bilder und sonstiges Drittmaterial unterliegen ebenfalls der genannten Creative Commons Lizenz, sofern sich aus der Abbildungslegende nichts anderes ergibt. Sofern das betreffende Material nicht unter der genannten Creative Commons Lizenz steht und die betreffende Handlung nicht nach gesetzlichen Vorschriften erlaubt ist, ist für die oben aufgeführten Weiterverwendungen des Materials die Einwilligung des jeweiligen Rechteinhabers einzuholen. Weitere Details zur Lizenz entnehmen Sie bitte der Lizenzinformation auf http://creativecommons.org/licenses/by/4.0/deed.de.

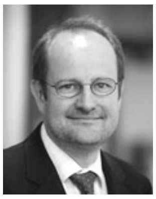

**Korrespondenzadresse:**
Prof. Dr. Harald Schwalbe
Institut für Organische Chemie und Chemische Biologie
Zentrum für Biomolekulare Magnetische Resonanz (BMRZ)
Universität Frankfurt a. M.
Max-von-Laue-Straße 7
D-60438 Frankfurt a. M.
schwalbe@nmr.uni-frankfurt.de
http://schwalbe.org.chemie.uni-frankfurt.de

Funding Open access funding durch dem SFB 902 Molekulare Mechanismen der RNA-basierten Regulation.

